# A Case Report and Brief Literature Review of Cerebrospinal Fluid Rhinorrhea in Granulomatosis With Polyangiitis: A Rare Complication

**DOI:** 10.7759/cureus.110035

**Published:** 2026-06-01

**Authors:** Swathi Balasubramanian, M.S.P.S Rajavel, Subramanian Nallasivan

**Affiliations:** 1 Medicine and Surgery, Velammal Medical College Hospital and Research Institute, Madurai, IND; 2 Otolaryngology, Velammal Medical College Hospital and Research Institute, Madurai, IND; 3 Rheumatology, Velammal Medical College Hospital and Research Institute, Madurai, IND

**Keywords:** anca-associated vasculitis, cerebrospinal fluid rhinorrhea, granulomatosis with polyangiitis, pr3-anca, small-vessel vasculitis

## Abstract

Granulomatosis with polyangiitis (GPA) is a necrotizing small-vessel vasculitis characterized by granulomatous inflammation and antineutrophil cytoplasmic antibody (ANCA) positivity. Sinonasal involvement is common, but cerebrospinal fluid (CSF) rhinorrhea is exceedingly rare. We present a rare case of a 44-year-old woman with established GPA who developed CSF rhinorrhea secondary to erosion of the cribriform plate. Her initial disease manifestations included episcleritis, septal perforation, atrophic turbinates, and saddle-nose deformity, with high PR3-ANCA titers and biopsy-proven small-vessel vasculitis. After achieving remission on corticosteroids and methotrexate, she presented four years later with watery nasal discharge that was triggered by bending forward. CT cisternography confirmed a CSF leak originating from a defect in the right anterior cribriform plate. She underwent successful endoscopic surgical repair using tensor fascia lata grafting, followed by immunosuppressive therapy and clinical improvement. This report highlights the potential for localized erosive disease in GPA despite apparent systemic remission and underscores the importance of timely recognition and multidisciplinary management.

## Introduction

Granulomatosis with polyangiitis (GPA), formerly called Wegener’s granulomatosis, is a necrotizing granulomatous small-medium vessel vasculitis, commonly associated with anti-neutrophil cytoplasmic antibodies (ANCA) [1]. It is characterized by necrotizing vasculitis of the upper and lower respiratory tracts and glomerulonephritis [2], though any organ system may be affected [3]. Ear, nose, and throat manifestations occur in 70-100% of cases [4], with common sinonasal symptoms including nasal obstruction, crusting rhinorrhea, hyposmia or anosmia, septal perforation, and saddle-nose deformity [4,5]. The nasal cavity and paranasal sinuses are the most frequently involved sites in the head and neck, affecting 85-100% of patients [4].

Immunologically, most patients with small-vessel vasculitis are ANCA-positive, showing either a cytoplasmic (c-ANCA) or perinuclear (p-ANCA) pattern on immunofluorescence. Quantitative testing for anti-proteinase 3 (anti-PR3) antibodies provides additional specificity for GPA. c-ANCA with anti-PR3 positivity is more commonly associated with systemic disease, whereas ANCA-negative cases may represent limited forms. Given its multisystem involvement, management often requires a multidisciplinary approach, including specialists in rheumatology, otorhinolaryngology, nephrology, ophthalmology, and other specialties [6].

GPA is rare, with a prevalence of 0.01% [7] and an annual incidence of 10-20 cases per million worldwide [8], typically affecting individuals aged 45-60 years, with no gender predilection. In Asia, the incidence ranges from 0.37 to 2.1 cases per million [9]. Cerebrospinal fluid (CSF) leak results from a communication between the skull base and the dura mater. In patients with GPA, this may be facilitated by reduced blood supply to the skull base and sinonasal mucosa [8]. CSF leak is an extremely rare complication of GPA, with few cases reported worldwide. To our knowledge, this represents the first reported case in India. We report a rare case of CSF rhinorrhea in a patient with GPA and review the relevant literature to emphasize the importance of early recognition and multidisciplinary management of this uncommon complication. Only a few cases have been reported in the literature, highlighting the rarity and clinical significance of this condition.

## Case presentation

A 44-year-old female presented in 2018 with complaints of a painful, red right eye, a blocked nose, and intermittent fever. She had no cough, weight loss, rash, or joint symptoms. Specialist evaluation revealed episcleritis, septal perforation, atrophic turbinates, and a saddle-nose deformity (Figure 1).

**Figure 1:**
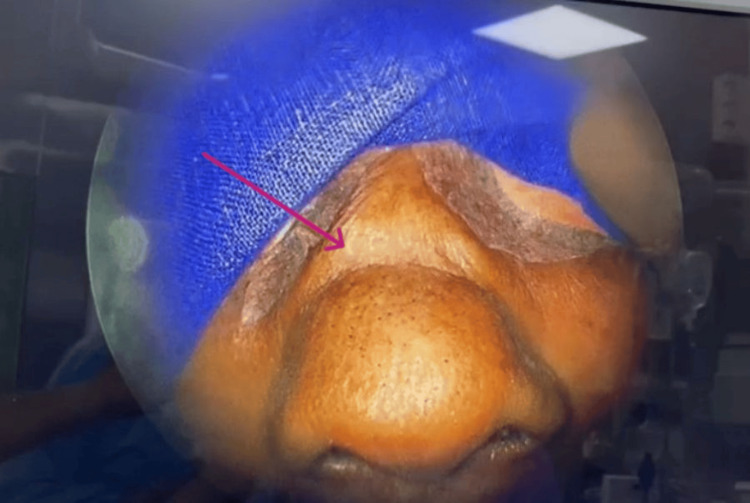
Frontal clinical photograph of the patient The image demonstrates a saddle-nose deformity (arrow) characterized by collapse of the nasal dorsum and flattening of the nasal bridge. These findings reflect chronic destructive changes of the nasal cartilage and septum, which are characteristic complications of granulomatosis with polyangiitis

Laboratory investigations showed an erythrocyte sedimentation rate (ESR) of 52 mm/hr, a C-reactive protein (CRP) level of 9.6 mg/dL, and a hemoglobin of 8.6 g/dL. A c-ANCA pattern was identified by indirect immunofluorescence, with anti-PR3 antibody positivity (1:640) confirmed by enzyme-linked immunosorbent assay (ELISA).

CT of the paranasal sinuses demonstrated a large septal perforation. CT of the brain did not show any erosive disease, and the lung fields on CT chest were normal. The anemia was attributed to active vasculitis. The patient underwent functional endoscopic sinus surgery (FESS) and a biopsy of the nasal septum. Histopathology revealed subepithelial tissue with mixed inflammatory infiltrates comprising lymphocytes, plasma cells, neutrophils, and eosinophils. Small blood vessels showed neutrophilic infiltration with variable fibrinoid necrosis and red blood cell extravasation, findings suggestive of small-vessel vasculitis. She was diagnosed with GPA (Table 1).

**Table 1:** Comparison of the patient's laboratory parameters at initial presentation and at relapse PR3-ANCA refers to anti-proteinase 3 antibodies measured by immunoassay WBC: white blood cell count; ESR: erythrocyte sedimentation rate; CRP: C-reactive protein; PR3-ANCA: proteinase 3-antineutrophil cytoplasmic antibody

Parameter	2018 (initial presentation)	2022 (relapse/complication)	Reference range
Hemoglobin	8.6 g/dL	Not specified	12–15 g/dL
WBC count	Not specified	7,800 cells/cu mm	4,000–11,000 cells/cu mm
ESR	52 mm/hr	38 mm/hr	<20 mm/hr
CRP	9.6 mg/dL	12 mg/L	<5 mg/L
PR3-ANCA	1:640 (positive)	Negative	<20 AU/mL
Renal function	Normal	Normal	Creatinine: 0.6–1.2 mg/dL; urea: 15–40 mg/dL

Initial treatment with corticosteroids followed by methotrexate resulted in clinical remission. During 2019-2020, the patient remained on maintenance therapy with regular follow-up via teleconsultation due to coronavirus disease 2019 (COVID-19) lockdown restrictions and difficulty traveling. In 2021, she developed progressive right eye involvement with low intraocular pressure, which progressed to phthisis bulbi, indicating locally active disease despite systemic remission. Following the rheumatology consultation, treatment was escalated to cyclophosphamide in addition to corticosteroids. However, progressive ocular damage ultimately necessitated enucleation in 2022.

In 2022, the patient presented with a one-week history of watery nasal discharge on bending forward and headache, with high clinical suspicion of CSF rhinorrhea based on the characteristic presentation. Although β-2 transferrin testing was not performed, CT cisternography was undertaken as the definitive diagnostic modality and demonstrated a defect in the anterior right cribriform plate with active CSF leakage, thereby confirming the diagnosis and guiding surgical management. This was further supported by intraoperative findings (Figure 2). Laboratory evaluation at that time showed a white blood cell count of 7,800 cells/cu.mm, CRP of 12 mg/L, ESR of 38 mm/hr, and a normal urine analysis. ANCA was negative, and systemic evaluation was normal.

**Figure 2:**
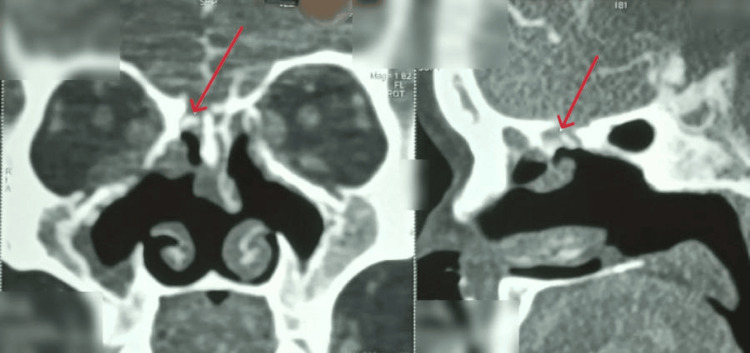
Axial CT cisternography The image (left) demonstrates a defect in the anterior part of the right cribriform plate (arrow). The arrow (right) highlights the site of bone erosion CT: computed tomography; CSF: cerebrospinal fluid

Surgical repair of the eroded cribriform plate was performed using a graft made of tensor fascia lata with Tisseel®. The patient received parenteral methylprednisolone, followed by oral mycophenolate. The postoperative course was uneventful, and she achieved remission. She is currently maintained on mycophenolate mofetil (Table 2).

**Table 2:** Clinical timeline of the disease course and management “Post-2022” refers to the follow-up period after surgical management of CSF leak ESR: erythrocyte sedimentation rate; CRP: C-reactive protein; PR3-ANCA: anti-proteinase 3 antineutrophil cytoplasmic antibodies; COVID-19: coronavirus disease 2019; CT: computed tomography; CSF: cerebrospinal fluid

Year/period	Clinical events	Investigations	Treatment	Outcome
2018	Red eye, nasal blockage, fever; ENT deformities	↑ESR, ↑CRP, PR3-ANCA positive, biopsy	Steroids followed by Methotrexate	Remission
2019–2020	Follow-up via local practitioner and teleconsultation (COVID-19 period)	Limited investigations available	Continued therapy	Stable (limited data)
2021	Progressive right eye involvement with low intraocular pressure	↑CRP	Steroids and cyclophosphamide	Progression to phthisis bulbi (enucleation performed in 2022)
2022	Watery nasal discharge, headache	CT cisternography showing a defect in the right cribriform plate with CSF leak	Surgical repair, along with steroids	Resolution of CSF leak
Post-2022	Follow-up	investigations stable	Mycophenolate mofetil	Remission

## Discussion

Our case report describes the occurrence of CSF leak due to erosion of the anterior part of the cribriform plate. A review of the literature is summarized in Table 3. Although the patient was under regular follow-up after diagnosis, the review was delayed during the COVID-19 pandemic. Her ANCA levels were within normal limits, suggesting remission. While disease flares are often associated with rising ANCA titers, this was not observed in our case. The presence of clear, watery nasal discharge should raise suspicion for CSF leak. Although β-2 transferrin testing was unavailable, CT cisternography, together with intraoperative confirmation, established the diagnosis with high confidence. CSF rhinorrhea is a rare complication of GPA, with only a limited number of cases reported worldwide. In this patient, it was most likely secondary to chronic structural damage to the cribriform plate.

**Table 3:** Reported cases of CSF rhinorrhea and skull base complications in GPA The table summarizes the previously reported cases of GPA complicated by skull base erosion and CSF rhinorrhea, including the present case. Clinical details are presented as reported in the original publications CSF: cerebrospinal fluid; GPA, granulomatosis with polyangiitis; PR3-ANCA: anti-proteinase 3 antineutrophil cytoplasmic antibody; CT: computed tomography; MRI: magnetic resonance imaging; CRP: C-reactive protein; ENT: ear, nose, and throat; GCS: Glasgow Coma Scale; EVD: external ventricular drain

Case	Initial presentation	Investigations/management	CSF leak manifestation and outcome
Case 1: reported in 2014, Caporali et al. [10]	A 67-year-old female presented with respiratory symptoms, a leg ulcer, and no active sinusitis. History of GPA for 9 years; had lung, ENT, and ocular involvement, left eye enucleation done	PR3-ANCA positive. CT thorax: bilateral ground-glass opacities. Treated with cyclophosphamide followed by corticosteroids and methotrexate, with subsequent administration of methylprednisolone resulting in clinical improvement	Presented with episodic headache, mild, fluctuant, altered mental status, and delayed verbal response. MRI brain demonstrated extensive pneumocephalus secondary to CSF leakage through multiple ethmoidal bone defects due to chronic GPA involvement of the anterior skull base
Case 2: reported in 2015, Akashi et al. [11]A	A 67-year-old male presented with chronic sinusitis and headache	MRI brain: cerebral pachymeningitis within the left hemisphere. Nasal endoscopy: large granulomatous lesions. PR3-ANCA positive. Elevated CRP. Initial clinical and radiological improvement was noted following prednisolone therapy	On day 19 of treatment, developed a severe headache and watery nasal discharge. CT brain: pneumocephalus. Surgical repair was performed on day 21 of glucocorticoid therapy
Case 3: reported in 2022, Sagi et al. [5]	An 80-year-old male presented with bilateral nasal congestion, crusting, and pain for x 6 weeks	Endoscopic nasal biopsy: significant necrosis and inflammation of the septum and left nasal sidewall, posterior septal perforation. PR3-ANCA positive. Pathology: necrotizing vasculitis with focal multinucleated giant cells, confirming GPA diagnosis. Lost to follow-up after initial diagnosis; re-presented 7 months later with disease progression - extensive sinonasal and nasopharyngeal bony destruction on imaging. Started on rituximab and corticosteroids	Later presented with complaints of nasal discharge. Nasal endoscopy: cribriform plate defect. Surgical repair performed using fascia lata graft (right thigh) and mucosal graft from the posterior wall of the left maxillary sinus
Case 4: present case (2022)	A 44-year-old female presented with a right painful red eye and nasal obstruction	PR3-ANCA positive (1:640 titre). CT sinus: large septal perforation. Nasal biopsy: small vessel vasculitis. Treated with corticosteroids and methotrexate; remission achieved	After 4 years, presented with complaints of watery nasal discharge on bending the head with headache. CT cisternography: erosion of the anterior right cribriform plate with CSF leak. Management: surgically corrected using a graft made of the tensor fascia lata. The postoperative course was uneventful, with the resolution of the CSF leak
Case 5: reported in 2024, Okita et al. [12]	A 33-year-old female presented with headache, nasal obstruction, and anosmia for 16 months. Examination: saddle nose deformity, septal perforation, rhinitis, sinusitis	MRI brain: hypertrophic pachymeningitis. PR3-ANCA positive. Nasal biopsy: nasal granulomatous lesions. Initial treatment with high-dose glucocorticoids and cyclophosphamide	Three months later, headaches worsened with diplopia and rhinorrhea. CT: septal perforation enlargement, skull base defects. MRI: hypertrophic pachymeningitis, posterior ethmoid sinus inflammation, and frontal lobe abscess. Management: treated with high-dose corticosteroids and rituximab; underwent craniotomy for defect closure and received long-term antibiotics for cerebral abscess
Case 6: reported in 2025, Lashkari S et al. [13]	An 80-year-old male. Past medical history: chronic nephropathy (unknown origin), severe allergic rhinosinusitis. Presented with right ophthalmoplegia, right diplopia, facial pain, and headache	Orbital MRI and CT: chronic rhinosinusitis with bony destruction extending to both orbits, involving the medial and inferior rectus muscles. Biopsy: inflammatory changes; ANCA negative. Initial treatment with prednisolone and methotrexate was later discontinued by the patient. Follow-up investigations: repeat orbital MRI: progressive bony destruction. Repeat biopsy: persistent inflammatory changes. Diagnosis: PR3-ANCA–negative GPA. Subsequent treatment with mycophenolate mofetil, followed by rituximab and azathioprine (intermittently discontinued due to non-adherence)	Lack of motivation, initiative, and planning for 1 month. Headache, dizziness, and paresthesia for 3 days. Drop in GCS while in hospital. Non-contrast CT head: erosion of the cribriform plate with spontaneous dural perforation causing frontal lobe pneumocephalus. Management: Initial placement of an EVD. Endoscopic endonasal repair using autologous fascia lata graft

A review of previously reported cases of CSF rhinorrhea in GPA demonstrates several consistent clinical patterns. Most patients presented with long-standing sinonasal disease, including chronic sinusitis, nasal crusting, and septal perforation, reflecting persistent upper airway involvement. Progressive erosion of the ethmoid and skull base, particularly the cribriform plate, appears to be the central pathological mechanism leading to CSF leakage. The underlying pathophysiology involves chronic granulomatous inflammation and necrotizing small-vessel vasculitis, resulting in progressive destruction of sinonasal structures. In advanced or long-standing disease, this process may extend to adjacent bony structures of the skull base, associated with serious intracranial complications, including pneumocephalus, cerebral abscess, and altered mental status in advanced cases.

While most patients were c-ANCA positive with elevated anti-PR3 antibodies, seronegativity does not exclude severe localized disease. CSF leaks have been observed both during active disease and after apparent remission or immunosuppressive therapy, suggesting that systemic disease control may not fully prevent ongoing localized destructive processes. Overall, these findings emphasize that GPA-related CSF rhinorrhea represents a rare but severe manifestation of persistent localized granulomatous and vasculitic inflammation with significant neurological morbidity. 

Corticosteroids remain the mainstay of treatment for active disease, with agents such as rituximab, methotrexate, and mycophenolate used as steroid-sparing therapies. Rituximab has been associated with favorable remission rates in recent times. In our case, the patient was unable to tolerate methotrexate on reintroduction and could not afford rituximab; therefore, mycophenolate mofetil was initiated. Despite negative ANCA levels, the disease appeared to remain locally active, necessitating a change in immunosuppressive therapy following surgical intervention to reduce the risk of persistent or recurrent disease. 

Although patients may achieve systemic remission and become ANCA-negative, the disease can remain locally active, causing erosion of the cribriform plate and ethmoid, resulting in CSF leak. ANCA titres do not always correlate with disease severity. Surgical intervention is currently the only proven method to correct these leaks. Timely identification by a physician or rheumatologist, followed by intervention by an ENT surgeon, is critical. A multidisciplinary approach has been shown to help these patients achieve remission. These complications may progress to serious intracranial sequelae such as pneumocephalus and cerebral abscess, as demonstrated in previously reported cases.

This case report is limited by its single-patient design and retrospective nature, and certain clinical details, such as the precise duration of symptoms before presentation and a complete pre-referral treatment history, were not fully available from the records. However, the presence of destructive sinonasal changes at initial presentation suggested longstanding localized disease before diagnosis. In addition, the small number of reported cases of CSF rhinorrhea in GPA limits robust comparative analysis, and the mechanisms underlying skull base erosion in this condition are not fully elucidated.

## Conclusions

This report highlights that ANCA-associated vasculitis requires regular follow-up, as locally destructive disease may occur even during apparent systemic remission, leading to serious complications such as CSF rhinorrhea. Management may require both surgical intervention and optimization of immunosuppressive therapy. Early recognition of warning symptoms, careful clinical evaluation, and timely multidisciplinary management are essential to prevent and manage such outcomes. The case also underscores the impact of interruptions in follow-up, as occurred during the COVID-19 pandemic in this patient, and the potential for significant morbidity, including vision loss, despite treatment.
